# Leptomeningeal collateral activation indicates severely impaired cerebrovascular reserve capacity in patients with symptomatic unilateral carotid artery occlusion

**DOI:** 10.1177/0271678X211024373

**Published:** 2021-06-10

**Authors:** Martina Sebök, Christiaan Hendrik Bas van Niftrik, Niklas Lohaus, Giuseppe Esposito, Mohamad El Amki, Sebastian Winklhofer, Susanne Wegener, Luca Regli, Jorn Fierstra

**Affiliations:** 1 Department of Neurosurgery, University Hospital Zurich, University of Zurich, Zurich, Switzerland; 2 Clinical Neuroscience Center, University Hospital Zurich, University of Zurich, Zurich, Switzerland; 3 Department of Neuroradiology, University Hospital Zurich, University of Zurich, Zurich, Switzerland; 4 Department of Neurology, University Hospital Zurich, University of Zurich, Zurich, Switzerland

**Keywords:** BOLD fMRI, cerebrovascular reactivity, collaterals, TCD, carotid artery occlusion

## Abstract

For patients with symptomatic unilateral internal carotid artery (ICA) occlusion, impaired cerebrovascular reactivity (CVR) indicates increased stroke risk. Here, the role of collateral activation remains a matter of debate, whereas angio-anatomical collateral abundancy does not necessarily imply sufficient compensatory flow provided. We aimed to further elucidate the role of collateral activation in the presence of impaired CVR. From a prospective database, 62 patients with symptomatic unilateral ICA occlusion underwent blood oxygenation-level dependent (BOLD) fMRI CVR imaging and a transcranial Doppler (TCD) investigation for primary and secondary collateral activation. Descriptive statistic and multivariate analysis were used to evaluate the relationship between BOLD-CVR values and collateral activation. Patients with activated secondary collaterals exhibited more impaired BOLD-CVR values of the ipsilateral hemisphere (p = 0.02). Specifically, activation of leptomeningeal collaterals showed severely impaired ipsilateral hemisphere BOLD-CVR values when compared to activation of ophthalmic collaterals (0.05 ± 0.09 vs. 0.12 ± 0.04, p = 0.005). Moreover, the prediction analysis showed leptomeningeal collateral activation as a strong independent predictor for ipsilateral hemispheric BOLD-CVR. In our study, ipsilateral leptomeningeal collateral activation is the sole collateral pathway associated with severely impaired BOLD-CVR in patients with symptomatic unilateral ICA occlusion.

## Introduction

The presence of impaired cerebrovascular reserve capacity (CVR) in patients with symptomatic unilateral internal carotid artery (ICA) occlusion is associated with increased stroke risk.^1–3^ The influence of collateral activation in this matter remains a matter of debate, since angio-anatomical collateral abundancy does not necessarily imply sufficient compensatory flow provided.^4–6^ Importantly, anatomically prominent collaterals may even reflect exhausted vasodilatation, and thereby impaired CVR. From the literature, it is known that up to 50% of symptomatic patients with ICA occlusion exhibit normal CVR,^7^ however the exact association between collateral circulation and impaired hemodynamic in those cases remains a matter of debate since there are important limitations in assessing the anatomical source of collateral flow status.

Collateral circulation is defined as alternate arterial routes that are activated when normal blood flow routes are insufficient. Using transcranial Doppler (TCD), the cerebral collateral vessel status can be noninvasively and inexpensively assessed with reliable evaluation of different intracranial blood flow patterns in real-time.^8^ For TCD measurements of the cerebral circulation, a distinction is made between primary and secondary collateral pathways.^9^ Primary collaterals are considered the anterior communicating artery (ACOM) and posterior communicating artery (PCOM) as part of the circle of Willis, whereas secondary collaterals include the ophthalmic artery and the more distal leptomeningeal collaterals.^10^ The latter is thought to be only recruited when the primary collaterals cannot be drawn upon or are insufficient to compensate the blood flow demand.^11^

Ideally, to best study the relationship between collateral activation and CVR, one would apply a multimodal imaging approach to both assess the primary and secondary collateral pathways as well as -*downstream*- brain tissue perfusion. Blood oxygenation-level dependent fMRI in combination with a standardized carbon dioxide (CO_2_) stimulus can yield quantitative CVR measurements at brain tissue level.^12,13^

We, therefore, studied the relationship between BOLD-CVR in the ipsilateral hemisphere of symptomatic patients with unilateral ICA occlusion and the activation of primary and secondary collateral pathways as examined with TCD, to determine which pathway is involved and activated in the presence of impaired BOLD-CVR.

## Materials and methods

The research ethics board of the Canton Zurich, Switzerland (Kantonale Ethikkommission Zurich; KEK-ZH-Nr. 2012-0427) approved this prospective ongoing cohort study, which is part of an interdisciplinary BOLD-CVR project in patients with symptomatic carotid artery disease. Written informed consent was obtained from each participant before inclusion. The study was conducted in accordance with the ethical standards as laid down in the 1964 Declaration of Helsinki and its later amendments.

### Patient inclusion

During the study period from February 2015 till February 2020, 78 patients with symptomatic carotid artery occlusion presented at the Clinical Neuroscience Center of the University Hospital Zurich, Switzerland and were subsequently screened for participation. Both, recently symptomatic patients (ischemic stroke or transient ischemic attack (TIA) or transient monocular blindness (TMB) in the last 30 days before inclusion) as well as formerly symptomatic patients (ischemic stroke or TIA or TMB at earlier time point) were included. Some patient data of this cohort were previously reported.^14^

The inclusion criteria were: (1) patients aged 18 years or above with symptomatic unilateral carotid artery occlusion, (2) exhibiting focal neurologic symptoms that are sudden in onset and ipsilateral to ICA occlusion, including one or more transient ischemic attacks, characterized by focal neurologic dysfunction or transient monocular blindness, or one or more minor (nondisabling) ischemic strokes^15^ and (3) who also underwent a TCD ultrasound examination within three weeks of the BOLD-CVR examination.

Excluded from the study were those patients with contraindications for MRI, intolerance for the soft plastic mask or for the applied CO_2_ stimulus during the BOLD-CVR fMRI examination. This was assessed under direct supervision of the subject by applying the CO_2_ stimulus outside the MRI system as a test-run. Patients who suffered from additional relevant vascular pathologies (>50% stenosis and/or occlusion) involving the anterior cerebral artery (ACA), middle cerebral artery (MCA), posterior cerebral artery (PCA), patients with additional relevant (≥50%) contralateral ICA stenosis as well as bilateral steno-occlusive pathologies (such as Moyamoya disease) were excluded since the additional pathologies could stimulate relevant leptomeningeal collateralization. Also excluded were patients with ICA dissection.

### BOLD fMRI acquisition and processing

#### BOLD fMRI and CO_2_ stimulus

MRI data were acquired on a 3-tesla Skyra VD13 scanner (Siemens Healthineers, Forchheim, Germany) with a 32-channel head coil. BOLD fMRI parameters and a three-dimensional (3 D) T1-weighted Magnetization Prepared Rapid Acquisition Gradient Echo (MP RAGE) image was performed in the same way as published in our previous work.^13^

During the BOLD fMRI sequence, the carbon dioxide (CO_2_) stimulus was given with a computer controlled gas blender with prospective gas targeting algorithms (RespirAct™, Thornhill Research Institute, Toronto, Canada). The RespirAct™ allows for precise targeting of arterial partial pressure CO_2_ while maintaining normal levels of O_2_ (iso-oxia).^16^ During the CVR study, a standardized and controlled hypercapnic stimulus is applied (i.e. the patients´ CO_2_ was increased ∼10 mmHg above their resting CO_2_ value for 80 seconds).^13^

All the acquired raw BOLD fMRI volumes were transferred to an external computer and pre-processed with SPM 12 (Statistical Parameter Mapping Software, Wellcome Department of Imaging Neuroscience, University College of London, London, UK). The BOLD fMRI volumes were processed and aligned to the T1-weighted MP RAGE image as well as smoothed with a Gaussian Kernel (for more information, see Sebök et al., 2018, Methods).^13^

#### BOLD-cerebrovascular reactivity maps

The BOLD-CVR calculations were done according to a previously described analysis pipeline.^12^ The analysis included a voxel-wise temporal shifting for optimal physiological correlation of the BOLD signal and BOLD-CO_2_ time series. BOLD-CVR, defined as the percentage BOLD signal change/mmHg CO_2_, was then calculated from the slope of a linear least square fit of the BOLD signal time course to the CO_2_ time series over the range of the first baseline of 100 seconds, the step portion of the protocol (80 seconds) and the second baseline of 100 seconds on a voxel-by-voxel basis. During the baseline portions of the protocol, all subjects were clamped on their resting CO_2_ baseline. The extra BOLD fMRI volumes were acquired to allow for potential temporal shift.^17^

### Transcranial doppler ultrasound technique

TCD examinations including color coded intra- and extracranial, transorbital and transforaminal sonography were performed on an Accuson Siemens X2000 duplex scanner. The arterial segments were routinely analyzed according to standardized methods as previously published.^6^ Grading of a stenosis of the contralateral ICA was performed by duplex sonography according to the NASCET criteria.^18^ Intracranial stenosis was graded according to established and published criteria.^19^

### Assessment of MR angiographic circle of willis anatomy and TCD derived collateral circulation pathways

Two imaging techniques were used to assess the anatomical and functional collateral status:

1. TOF-MRA to classify the anatomy of the Circle of Willis and the number of available anatomical collaterals, and
2. TCD to identify presence of collateral activation of primary and secondary collateral pathways.

#### Circle of willis in TOF-MRA

Time-of-Flight (TOF)-magnetic resonance angiography (MRA) of the first MRI performed after hospital admission by diagnosis of unilateral symptomatic ICA occlusion was used to classify the anatomy of the Circle of Willis (CoW). The following vessels were assessed as present or not present: ACOM, A1-segments of anterior cerebral artery, PCOM, P1-segment of posterior communicating artery (PCA). Furthermore, the configuration of a full (no PCA-P1-segment assessable) or partial (PCOM larger in size than the PCA-P1-segment) fetal posterior communicating artery was assessed. If TOF-MRA was not fully conclusive, contrast enhanced MRA (only if performed in the same MRI-investigation) was used to determine CoW anatomy. For determination of collateral status of the patient, the ophthalmic artery was assessed for absence or presence of flow signal on TOF-MRA.

The CoW in TOF-MRA images were evaluated independently by two neuroradiologists (S.Wi. with 10 years of experience and N.L. with two years of experience) blinded to the clinical data. Disagreements were settled by consensus.

#### TCD examination of collateral pathways

All TCD measurements were performed in clinical routine by an experienced vascular neurologist or a resident neurologist in the presence of an experienced vascular neurologist. We collected the results through medical chart review and evaluation of the saved ultrasound images from the TCD investigation. The identification of the four main collateral pathways that can be assessed with TCD, was done as follows.^6,20,21^

1. ACOM: Reversed flow direction in the first (A1) segment of the ACA ipsilateral to the occluded ICA
2. PCOM: cerebral blood flow increase of > 50% in the ipsilateral first (P1) segment of the PCA compared with the contralateral side
3. Ophthalmic artery (OA): reversed flow in the periorbital arteries along with an internalized flow profile
4. Leptomeningeal (LPM) collateral activation: flow increase of > 30% in the ipsilateral second (P2) segment of the posterior cerebral artery (PCA) compared with the contralateral P2 segment (Figure 2(a) and (b)). Important to mention is that leptomeningeal collateral activation for TCD implies the leptomeningeal collateral pathway supplied by the posterior circulation.

#### Activated collateral pathways

Using the number of available anatomical collaterals and number of activated collaterals as assessed by Doppler TCD investigation, we demonstrate the percentage (%) of activated collaterals as the quotient between activated collaterals in TCD and possible collaterals in anatomical images. Here, for the number of possible collaterals we used the data for ACOM and PCOM from TOF MRA images, for ophthalmic artery the measured flow in Doppler TCD verified for presence of possible collateral since the ophthalmic artery identification in TOF MRA is due to anatomical conditions limited.^22^ If no ophthalmic artery flow was measurable in TCD and no ophthalmic artery identifiable in TOF MRA, we counted as no anatomical possibility for ophthalmic collateral. Leptomeningeal collaterals were in all patients considered as anatomically possible.

Activation of maximal four collateral pathways (ACOM, PCOM, ophthalmic and leptomeningeal collaterals) is possible. However, after anatomical correction there are also patients with only three or two anatomically possible collaterals. Therefore, the quotient between activated collaterals in TCD and possible collaterals in anatomical images is always calculated for the corrected number of anatomical collaterals. For example: in patient with three possible collaterals as seen in TOF MRA and activation of two of those collaterals in TCD, the percentage of activated collaterals is calculated as: 2/3 * 100% = 67%.

### DWI masking

Each infarct volume were manually outlined on diffusion weighted imaging (DWI) with apparent diffusion coefficient (ADC) sequences by a board certified neuroradiologist (S.Wi.) with 10 years of experience using MRIcro software (version 1.4; Chris Rorden). The acute phase sequences were used for infarct volume analysis. When multiple ischemic infarct lesions were present, the cumulative infarct volume would represent the sum of all the lesions. Based on the T1-weighted imaging properties, infarct volumes were binarized, transformed into Montreal Neurological Institute space using SPM, and calculated as the sum of the number of voxels (in ml).

### Statistical analysis

All statistical analyses were carried out using R (R Core Team, 2020).^23^ All continuous variables are reported as mean ± SD. Categorical ordinal variables are presented as median (interquartile range (IQR)), whereas dichotomous variables are shown as frequency (%). Continuous variables were compared by an independent Student´s 2-tailed t test, where p < 0.05 was considered statistically significant. ANOVA was used to calculate the between group differences. Prediction analysis to evaluate the relationship between activated collaterals and BOLD-CVR values of the ipsilateral hemisphere were done using univariate and multivariate analysis and evaluated using the R Squared (R^2^) diagnostic. The predictive quality was assessed using the Akaike Information Criterion (AIC). To avoid Type I error after multiple testing, Bonferroni correction was applied.^24^

## Results

### Study population characteristics

**Figure 1.**
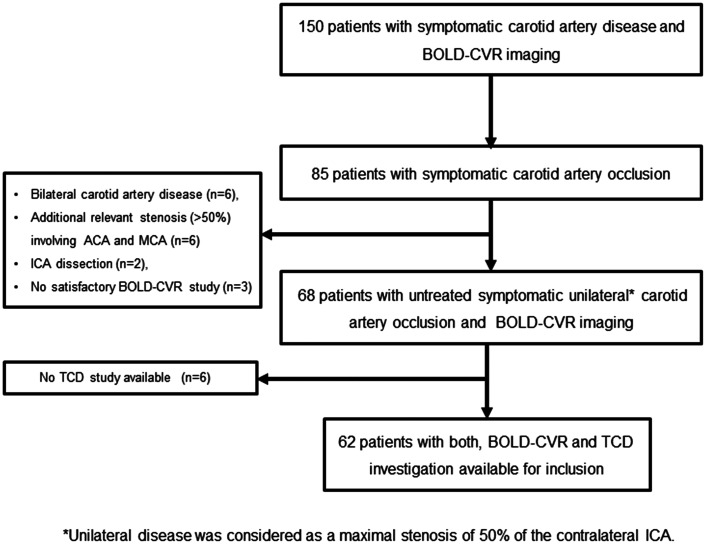
Study flow chart. From 150 patients with symptomatic carotid artery disease included in our prospective BOLD-CVR database, 85 patients were diagnosed with symptomatic carotid artery occlusion. Out of them, 68 patients presented with untreated symptomatic unilateral (maximal stenosis of 50% of the contralateral ICA) carotid artery occlusion and underwent a BOLD-CVR study and were available for inclusion in this prospective cohort study. In six patients out of 68, no TCD study was available. In the final analysis, 62 patients with untreated unilateral symptomatic ICA occlusion, who underwent both, BOLD-CVR and TCD investigation, were included. BOLD: blood oxygen-level dependent; CVR: cerebrovascular reactivity; ICA: internal carotid artery; TCD: transcranial Doppler.

During the study period, 85 patients with symptomatic carotid artery occlusion, who presented at the Clinical Neuroscience Center of University Hospital Zurich, Switzerland were screened for participation in this study. Of these, 68 patients were diagnosed with untreated symptomatic unilateral ICA occlusion and underwent BOLD-CVR investigation. Since in six patients no TCD study was available, 62 participants were considered for further analysis. A flow-chart illustrating patient screening and inclusion can be reviewed in Figure 1.

Mean age of the included patients was 65.6 ± 11.6 years, 14 (22.6%) were female. All patients presented with permanent or transient ischemic clinical symptoms prior to imaging diagnosis of new ICA occlusion. Forty-six patients presented with ischemic stroke, 13 with TIA and 3 with TMB. The mean infarct volume in patients presenting with ischemic stroke was 6.78 ± 9.88 ml. The median time between the neurological symptoms (TIA, TMB or ischemic stroke) and BOLD-CVR investigation was five days (interquartile: 17 days). The median time between the neurological symptoms and TCD study was two days (interquartile: five days). The patients had the following cerebrovascular risk factors: 40 (64.5%) patients had hypertension, 12 (19.4%) had diabetes, 30 (48.4%) hypercholesterolemia, four (6.5%) patients carried a positive family history for cerebral ischemic events and 13 (21.0%) had reported obesity. Thirty (48.4%) patients were active or previous long-term smokers.

The baseline values of TCD assessment of flow (peak systolic velocity and end diastolic velocity) for relevant intracranial arteries measured ipsilateral and contralateral to ICA occlusion can be reviewed in Supplementary Table 1.

### Cerebrovascular reserve capacity and collateral circulation status in patients with symptomatic carotid occlusion

**Figure 2.**
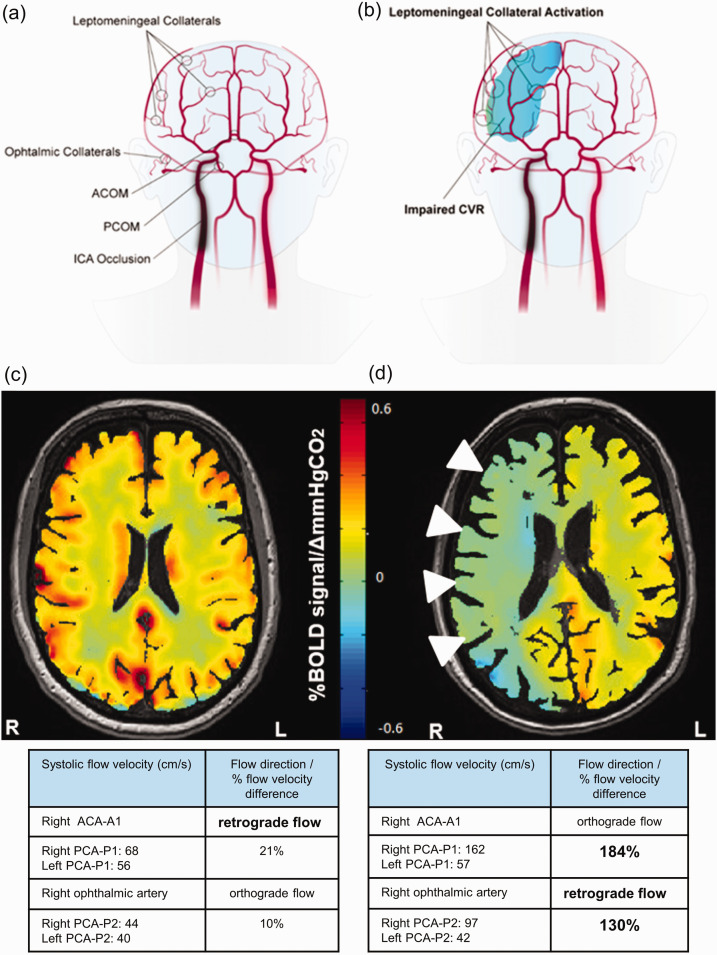
Schematic representation of collateral pathways and exemplary BOLD-CVR images of two patients with right ICA occlusion. (a) The figure shows the available four collateral pathways (two primary collaterals and two secondary collaterals) in patients with unilateral ICA occlusion. The two primary collaterals, which are part of the Circle of Willis, are ACOM and PCOM. Activation of ACOM is detected as reversed flow direction in the first (A1) segment of the anterior cerebral artery ipsilateral to the occluded ICA. Activation of PCOM is identified as cerebral blood flow increase of > 50% in the ipsilateral first (P1) segment of the posterior cerebral artery compared with the contralateral side. The two TCD-derived secondary collaterals are ophthalmic and leptomeningeal collaterals supplied by the posterior circulation. Activation of ophthalmic collaterals is detected as reversed flow in the periorbital arteries and activation of leptomeningeal collaterals as flow increase of > 30% in the ipsilateral second (P2) segment of posterior cerebral artery compared with the contralateral P2 segment. (B) The figure shows activation of leptomeningeal collaterals on the surface of the ipsilateral (=side of ICA occlusion) hemisphere with impaired (=paradox) CVR in this hemisphere. (c) BOLD-CVR image of a 48-years old patient with occlusion of right ICA showed preserved CVR in the territory of the occluded vessel. The TCD examination showed only primary activation through ACOM with reversed flow direction in the right ACA-A1 segment. (d) BOLD-CVR image of 85-years old patient with occlusion of right ICA showed impaired CVR in the territory of the occluded vessel. The TCD examination showed: 1) right sided PCOM activation with 184% increase of SFV of the right PCA-P1 segment compared to the contralateral PCA-P1, 2) reversed flow in the right ophthalmic artery indicating right sided ophthalmic activation, and 3) activation of leptomeningeal collateral pathways supplied by the posterior circulation on the ride side with 130% increase of SFV of the right PCA-P2 segment compared to the contralateral PCA-P2. ACA-A1: first segment of anterior cerebral artery; ACOM: anterior communicating artery; BOLD: blood oxygenation-level dependent; CVR: cerebrovascular reactivity; ICA: internal carotid artery; PCA-P1: first segment of posterior cerebral artery; PCA-P2: second segment of posterior cerebral artery; PCOM: posterior communicating artery; SFV: systolic flow velocity; TCD: transcranial Doppler.

**Figure 3.**
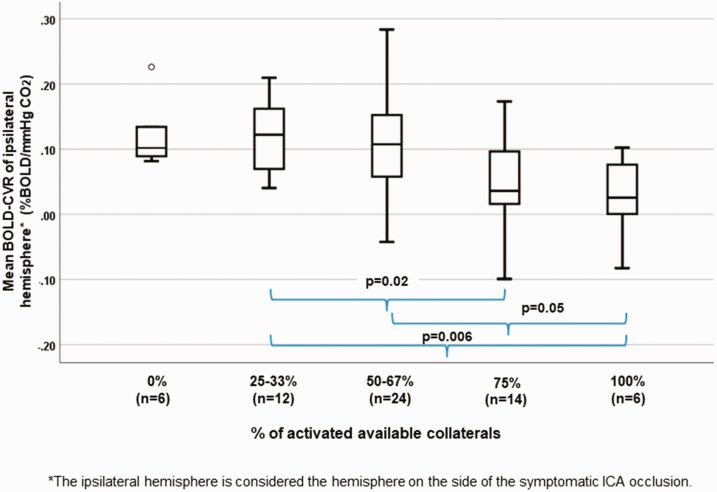
Correlation between percentage of activated available collaterals and mean ipsilateral hemisphere BOLD-CVR values. Box-whisker plots show the correlation between the percentages of activated available collaterals and mean BOLD-CVR values of the ipsilateral hemisphere. Patients with more activated collaterals exhibit significantly lower CVR values: patients with 75% and 100% of activated available collaterals have significantly lower mean BOLD-CVR of ipsilateral hemisphere as compared to patients with activated 25-33% of available collaterals; patients with 100% collateral activation exhibit significantly lower CVR values compared to patients with 50-67% collateral activation. Activation of maximal four collateral pathways (ACOM, PCOM, ophthalmic artery, and leptomeningeal collateral pathways supplied by the posterior circulation) is possible. However, after anatomical correction there are also patients with only three or two anatomically possible collaterals. Therefore, the quotient between activated collaterals in TCD and possible collaterals in anatomical images is always calculated for the corrected number of anatomical collaterals. For example: in patient with three possible collaterals as seen in TOF MRA and activation of two of those collaterals in TCD, the percentage of activated collaterals is calculated as: 2/3 * 100% = 67%. Patients with activation of 1/4 and 1/3 of anatomically available collaterals form the 25-33% group and patients with activation of 1/2 and 2/3 of anatomically available the 50-67% group. Note: The box of box-whisker plots represents the median value with interquartile range (25th to the 75th percentile). The upper and lower whiskers represent values outside the middle 50% (i.e., the values below 25th and above 75th percentile). BOLD: blood oxygen-level dependent; CVR: cerebrovascular reactivity.

Table 1 presents the baseline BOLD-CVR values and TCD-derived collateral vessel status of 62 patients with symptomatic unilateral carotid artery occlusion. Exemplary BOLD-CVR images and TCD based collateral status evaluation of two patients with right sided ICA occlusion can be reviewed in Figure 2. Calculating the quotient between number of affected collaterals in TCD and number of for activation possible collaterals (as seen in TOF MRA), six (9.7%) of patients had 0% collateral activation as identified by TCD examination according to the above-mentioned criteria (see 2.4.2 Methods section).Twelve (19.4%) patients had 25-33% activation, 24 (38.7%) patients had 50-67% activation, 14 (22.6%) patients had 75% activation and six (9.7%) patients had 100% activation. The relationship between % of collateral activation and mean BOLD-CVR values of ipsilateral hemisphere is demonstrated in Figure 3.

**Table 1.** BOLD-CVR values and collateral vessel status of patients with symptomatic unilateral carotid artery occlusion.

Variable	Symptomatic unilateral ICA occlusion cohort(n = 62)
**Hemodynamic BOLD-CVR data (mean ± SD)**	
Mean BOLD-CVR whole brain	0.11 ± 0.07
Mean BOLD-CVR grey matter	0.13 ± 0.08
Mean BOLD-CVR white matter	0.07 ± 0.06
Mean BOLD-CVR ipsilateral^a^ hemisphere	0.09 ± 0.08
Mean BOLD-CVR contralateral hemisphere	0.14 ± 0.07
**TCD collateral vessel status data, n (%)**	
ACOM collateral	42 (67.7)
PCOM collateral	12 (19.4)
Ophthalmic collateral	36 (58.1)
Leptomeningeal collateral	27 (43.5)
Activation of primary collaterals	49 (79)
Activation of secondary collaterals (with/without primary)	46 (74.2)

^a^The ipsilateral hemisphere is considered the hemisphere on the side of the symptomatic ICA occlusion.

Significantly lower mean BOLD-CVR value of the ipsilateral hemisphere was found in patients with 75% of available collaterals activated compared to those with activated 25-33% of available collaterals (mean BOLD-CVR ± SD: 75%: 0.05 ± 0.07 vs. 25-33%: 0.12 ± 0.05, p = 0.02). Even better pronounced is the BOLD-CVR difference of ipsilateral hemisphere between patients with activated 100% available collaterals and 25-33% available collaterals (mean BOLD-CVR ± SD: 0.02 ± 0.06 vs. 0.12 ± 0.05, p = 0.006). Markedly impaired BOLD-CVR of ipsilateral hemisphere was seen also in patients with 100% collateral activation compared to patients with 50-67% collateral activation (mean BOLD-CVR ± SD: 0.02 ± 0.06 vs. 0.10 ± 0.09, p = 0.05) (Figure 3). The between groups difference in ipsilateral BOLD-CVR values by one-way ANOVA is p = 0.03.

### Impact of activation of primary collaterals on patients´ hemodynamic status

Patients with ACOM activation (42 patients) showed higher BOLD-CVR values in the ipsilateral hemisphere as compared to patients without ACOM activation (mean BOLD-CVR ± SD: 0.10 ± 0.08 vs. 0.06 ± 0.08, p = 0.07). Interestingly, patients with PCOM activation (14 patients) showed a clear significant BOLD-CVR decrease in the ipsilateral hemisphere as opposed to patients without PCOM activation (mean BOLD-CVR ± SD: 0.04 ± 0.08 vs. 0.10 ± 0.07, p = 0.006).

### Impact of activation of secondary collaterals on patients´ hemodynamic status

Patients with activated secondary collaterals (with or without primary collaterals) exhibited significantly lower BOLD-CVR values of ipsilateral hemisphere as compared to patients with only primary collaterals activation (mean BOLD-CVR ± SD: 0.07 ± 0.08 vs. 0.13 ± 0.05, p = 0.02).

Patients with activation of ophthalmic collaterals (36 patients) showed significantly lower BOLD-CVR values in the ipsilateral hemisphere as compared to patients without ophthalmic activation (mean BOLD-CVR ± SD: 0.07 ± 0.07 vs. 0.11 ± 0.08, p = 0.05). Activation of leptomeningeal collaterals is associated with even lower BOLD-CVR values – mean BOLD-CVR of ipsilateral hemisphere of patients without leptomeningeal collaterals vs. patients with leptomeningeal collaterals: 0.04 ± 0.09 vs. 0.12 ± 0.04, p < 0.001.

**Figure 4.**
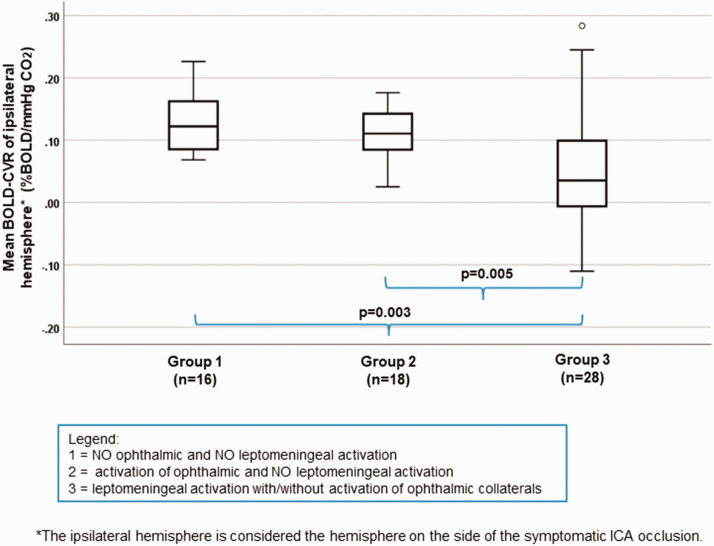
Correlation between different stages of secondary collaterals activation and mean ipsilateral hemisphere BOLD-CVR values. Box-whisker plots show the correlation between the three groups of patients with different secondary collaterals activation status and BOLD-CVR values of ipsilateral hemisphere. Patients with activated leptomeningeal collateral pathways supplied by the posterior circulation with/without activation of ophthalmic collaterals (group 3) exhibit significantly lower mean BOLD-CVR values of ipsilateral hemisphere compared to patients without any secondary collaterals (group 1) (mean BOLD-CVR ± SD: 0.05±0.09 vs. 0.13±0.05, p=0.003) as well as compared to patients with activation of only ophthalmic collaterals (group 2) (mean BOLD-CVR ± SD: 0.05±0.09 vs. 0.12±0.04, p=0.005). No difference is BOLD-CVR values is discernible between patients without any activated secondary collaterals and between patients with only ophthalmic activation. The between groups difference for ipsilateral BOLD-CVR values by one-way ANOVA is p=0.001. Note: The box of box-whisker plots represents the median value with interquartile range (25th to the 75th percentile). The upper and lower whiskers represent values outside the middle 50% (i.e., the values below 25th and above 75th percentile). ANOVA: analysis of variance; BOLD: blood oxygen-level dependent; CVR: cerebrovascular reactivity; SD: standard deviation.

By studying the impact of secondary collaterals activation, patients were divided into three groups: group 1 without activation of ophthalmic and leptomeningeal collaterals, group 2 with activation of ophthalmic and without activation of leptomeningeal collaterals and group 3 with activation of leptomeningeal collaterals and with/without activation of ophthalmic collaterals. A significant lower mean BOLD-CVR value of ipsilateral hemispheres is seen in patients with activated leptomeningeal collaterals (group 3) compared to those without any secondary collaterals (group 1) (mean BOLD-CVR ± SD: 0.05 ± 0.09 vs. 0.13 ± 0.05, p = 0.003) as well as between group 2 (activation of ophthalmic collaterals without leptomeningeal activation) and group 3 (activation of leptomeningeal collaterals with/without ophthalmic collaterals): mean BOLD-CVR ± SD: 0.05 ± 0.09 vs. 0.12 ± 0.04, p = 0.005 (Figure 4). The between groups difference for ipsilateral BOLD-CVR values by one-way ANOVA is p = 0.001.

### Effect of PCOM and leptomeningeal collateral activation on CVR

**Figure 5.**
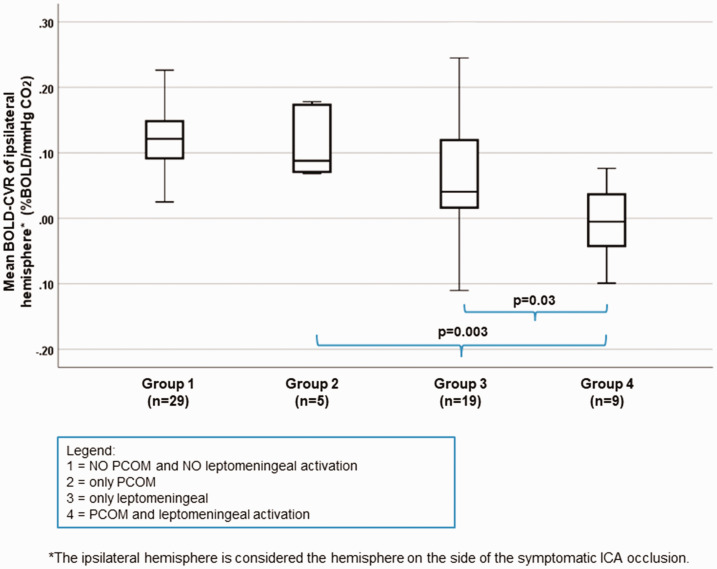
Correlation between different stages of PCOM and leptomeningeal collaterals activation and mean ipsilateral hemisphere BOLD-CVR values. Box-whisker plots show the correlation between the groups of patients with different activation status of PCOM and leptomeningeal collateral pathways supplied by the posterior circulation and BOLD-CVR values of ipsilateral hemisphere. Patients with activated both, PCOM and leptomeningeal collaterals (group 4) exhibit significantly lower mean BOLD-CVR values of ipsilateral hemisphere compared to patients with only PCOM activation (group 2) (mean BOLD-CVR ± SD: -0.01±0.06 vs. 0.12±0.06, p=0.003) and patients with only leptomeningeal activation (group 3) (mean BOLD-CVR ± SD: mean BOLD-CVR ± SD: -0.01±0.06 vs. 0.07±0.09, p=0.03). The between groups difference for ipsilateral BOLD-CVR values by one-way ANOVA is p<0.001. Note: The box of box-whisker plots represents the median value with interquartile range (25th to the 75th percentile). The upper and lower whiskers represent values outside the middle 50% (i.e., the values below 25th and above 75th percentile). ANOVA: analysis of variance; BOLD: blood oxygen-level dependent; CVR: cerebrovascular reactivity; PCOM: posterior communicating artery; SD: standard deviation.

Since patients with PCOM activation (independent of activation status of other possible collaterals) exhibit a mean ipsilateral hemisphere BOLD-CVR value of 0.04 ± 0.08 and patients with activation of leptomeningeal collaterals (independent of activation status of other possible collaterals) a mean ipsilateral hemisphere BOLD-CVR value of 0.04 ± 0.09, to demonstrate the true impact of PCOM and LPM collaterals, the patients were divided into four groups: group 1 without activation of PCOM and leptomeningeal collaterals, group 2 with activation of only PCOM collaterals, group 3 with activation of only leptomeningeal collaterals and group 4 with activation of both, PCOM and LPM collaterals. No BOLD-CVR difference is seen between group 2 and 3, but a significantly lower BOLD-CVR in group 4 (activated both, PCOM and LPM collaterals) as compared to group 2 (mean BOLD-CVR ± SD: −0.01 ± 0.06 vs. 0.12 ± 0.06, p = 0.003) as well as compared to group 3 (mean BOLD-CVR ± SD: −0.01 ± 0.06 vs. 0.07 ± 0.09, p = 0.03). The between groups difference for ipsilateral BOLD-CVR values by one-way ANOVA is p < 0.001. (Figure 5).

Investigation of quantitative flow velocity values showed interesting results: the mean quantitative systolic PCA-P1 flow velocity did not differ between patients in group 2 and 4 (116 ± 80cm/s vs. 120 ± 44, p = 0.91). This indicates that the lower BOLD-CVR of ipsilateral hemisphere found in patients with PCOM activation is primarily driven by leptomeningeal activation.

Using prediction analysis to determine the independent effect of each collateral pathway on BOLD-CVR of the ipsilateral hemisphere, in the univariate analysis PCOM collaterals (R^2^ = 0.11, p = 0.005) and leptomeningeal collaterals (R^2^ = 0.21, p < 0.001) showed a significant association with BOLD-CVR, while ophthalmic and ACOM activation did not. In the multivariate analysis (R^2^ = 0.30), leptomeningeal activation (p = 0.003) remained the sole collateral pathway independently predict the BOLD-CVR of the ipsilateral hemisphere (PCOM activation: p = 0.06, ophthalmic activation: p = 0.09).

## Discussion

In this study, we examined symptomatic unilateral ICA occlusion patients with BOLD-CVR and TCD to determine which collateral pathway activation have influence on BOLD-CVR by evaluating primary collateral activation (ACOM and PCOM activation) and secondary collateral activation (ophthalmic artery and leptomeningeal artery activation). Our data demonstrate that only activation of leptomeningeal collateral pathways as measured by the difference of >30% between the ipsilateral and contralateral PCA-P2 flow velocity was associated with impaired BOLD-CVR in the ipsilateral hemisphere in patients with symptomatic unilateral ICA occlusion. Although the activation of the ophthalmic artery and PCOM activation were associated with a more impaired BOLD-CVR, this association was confounded by underlying leptomeningeal collateral activation. Using a prediction analysis to determine the independent effect of each collateral pathway on BOLD-CVR of the ipsilateral hemisphere, leptomeningeal collateral activation supplied by the posterior circulation were shown to be independent predictor of ipsilateral hemisphere BOLD-CVR.

### Collateral status of patients with carotid occlusion

Patients with ICA occlusion and an impaired cerebrovascular reactivity are thought to have an increased stroke risk.^1–3^ The rate of subsequent recurrent stroke in patients with ICA occlusion has been estimated 2.4-7% per year,^25,26^ but is significantly higher in patients with impaired reactivity.^27,28^ Therefore, an identification of patients with symptomatic ICA occlusion, exhausted hemodynamic status and insufficient collateral status plays a key role in treatment of those patients. Moreover, a recent study also found that presence of hemodynamic impairment significantly shortened event-free survival during the follow-up in patients with symptomatic carotid artery occlusion.^29^ Several previous studies linked the carotid occlusion in patients with symptomatic cerebral ischemia with hypoperfusion and in addition, exhaustion of compensatory reserve of the cerebral circulation.^30–32^ Severe hemodynamic failure has been linked with recruitment of collateral pathways, especially with leptomeningeal collaterals.^14,33,34^ Many noninvasive imaging techniques are standardly used to evaluate primary and secondary collateral circulation.^35,36^ However, digital subtraction angiography (DSA) remains the gold standard for structural and functional evaluation of collaterals,^25,37^ in everyday clinical practice TCD is often used as method of choice to assess collateral sufficiency.

Our results show an interesting pattern of collateral activation: patients with ICA occlusion and activation of only primary collateral pathways present with preserved BOLD-CVR, which is congruent with the earlier findings in the literature.^38,39^ Activation of secondary collaterals resulted in a decrease in BOLD-CVR, however this was only the case if leptomeningeal activation was present. Retrograde flow in the ophthalmic artery alone did not result in impaired BOLD-CVR. Previous studies showed conflicting results regarding impact of secondary collaterals: while some studies linked the activation of secondary collaterals to impaired cerebral vasoreactivity,^11,21^ others suggested that not the type but the number of activated collateral pathways indicates a higher risk of stroke recurrence in patients with carotid artery occlusion.^6,38,40^

Connolly et al.^41^ assessed the activation of primary and secondary collaterals in patients with symptomatic ICA occlusion using TCD, and showed that ACOM collateral was most frequently activated, followed by ophthalmic, the PCOM and the leptomeningeal collaterals. In our cohort, likewise, ACOM collateral was most frequently activated followed by ophthalmic. Contrary, PCOM in our cohort was activated only in 20%, clearly less than leptomeningeal collaterals. This could be due to the larger cerebral blood flow difference necessary to be present for PCOM activation (>50%) as compared to leptomeningeal activation (>30%) – see also Methods section. However, these cut-offs are accepted as clinical standards. Secondly, the TCD examination, which is operator dependent, has to be considered.

### Pathways of secondary collateral activation

The activation of secondary collaterals in addition to primary collaterals was already in previous studies related to compromised – *exhausted* – hemodynamic status.^6,11,14^ A negative flow reactivity is significantly associated with a dependence on leptomeningeal collaterals and implies a state of maximal hemodynamic compromise. In 1994, Smith et al.^42^ in patients with symptomatic cerebrovascular disease who underwent angiography and xenon-enhanced computed tomographic cerebral blood flow studies before and after acetazolamide found a significant association between negative flow reactivity and a dependence on leptomeningeal collaterals indicating a state of maximal hemodynamic compromise.

A recent study by Hartkamp et al.^33^ supports our findings too, as the recruitment of secondary collaterals was associated with severe hemodynamic impairment. In their cohort, secondary collateral flow occurred only in patients with symptomatic ICA occlusion, who also had severe hemodynamic impairment of the affected hemisphere. Based on these findings they hypothesized that the occurrence of secondary collaterals is due to critically insufficient primary collateral redistribution via the Circle of Willis. Comparably, a recent study using perfusion weighted imaging and quantitative T2* mapping based assessment of leptomeningeal collaterals concluded that increased leptomeningeal collateral supply cannot necessarily be regarded as a sign of effective compensation in patients with high-grade steno-occlusive vasculopathy.^43^ Our results confirm this hypothesis. Moreover, with significantly lower BOLD-CVR values in patients with activated leptomeningeal collaterals (independent of ophthalmic activation) as compared to patients with activated ophthalmic collaterals without leptomeningeal collaterals, a clear priority in activation of secondary collaterals is detectable. Namely, ophthalmic collaterals activate first after the collateral flow through Circle of Willis is not sufficient, and leptomeningeal as the last one in the case of exhausted hemodynamic as a sort of tertiary collateral pathway.

### Impaired CVR and leptomeningeal activation as indicators of exhausted cerebral hemodynamic status in symptomatic carotid artery occlusion

Our results confirm the previously reported finding that activation of leptomeningeal collaterals^33,43^ suggests a compromised hemodynamic status. A similar finding was reported by Strother et al.^44^ in patients with Moyamoya disease, who find that the degree of leptomeningeal collateral supply reflects the disease severity. A significant association between activation of leptomeningeal collaterals with/without PCOM as well as between LPM with/without ophthalmic collaterals and impaired (even negative) BOLD-CVR of ipsilateral hemisphere in our cohort, confirms the link between activation of leptomeningeal collaterals and exhausted BOLD-CVR. Moreover, we have shown that the mean quantitative systolic PCA-P1 flow velocity did not differ between patients with only PCOM activation and patients with both, PCOM and leptomeningeal collateral activation, indicating that the lower BOLD-CVR of ipsilateral hemisphere found in patients with PCOM activation is primarily driven by leptomeningeal activation.

Our prediction model showed that leptomeningeal collateral activation supplied by the posterior circulation is sole independently predictive of BOLD-CVR of the ipsilateral hemisphere. As flow velocity measured from TCD represents a combination of cerebral blood flow and vessel radius, the higher PCA-P2 flow velocity values may represent a functional stenosis rather than just an increased cerebral blood flow due to increased compensatory efforts for the need of the vascular bed.^6^ Both TCD defined activation of PCOM collaterals and leptomeningeal collaterals are based on a difference analysis between ipsilateral and contralateral PCA-P1 and PCA-P2 segment, respectively. The first risk of such a difference analysis is false positive, especially in patients with low contralateral flow velocity values. However, it is also known that unilateral vasculopathy can affect both hemispheres and in that case this difference analysis could result in false negative.^12,45^ Schneider et al.^6^ also found a bilateral increase in PCA-P2 flow in patient with recurrent stroke. It is expected, therefore, that more patients do have a functional PCA-P1 and PCA-P2 stenosis and to have a quantitative cut-off point would be more optimal to define PCOM and leptomeningeal activation as the difference analysis. However, the clear difference between patients with and without functional stenosis of the PCA-P2 does show its clinical value as an easy parameter to evaluate.

### Future considerations & clinical implications

To have a specific TCD parameter available, i.e. leptomeningeal collateral activation, may further support clinicians in screening a wide population of patients with carotid artery occlusion and identify those, whom may need a further cerebral hemodynamic workup, for instance with BOLD-CVR. Specifically, patients with ICA occlusion and TCD identified leptomeningeal activation may be those who need further hemodynamic work-up with BOLD-CVR (or any other imaging modality that can assess cerebral hemodynamics) to detect those suffering from hemodynamic failure. Secondly, this may also improve patient selection for revascularization strategies. This is important not only to prevent recurrent ischemic strokes but also to avoid a cognitive deterioration in the presence of carotid occlusion, especially when associated to impaired CVR.^46,47^ For future studies, however, it is important to mention that a validation of the present findings using a sensitivity and specificity analysis would be of utmost clinical importance.

### Limitations

The study represents single center-based findings in a cohort of patients with symptomatic unilateral ICA occlusion. A selection bias was introduced to the study since especially patients with internal carotid occlusion, who were suspected of having hemodynamic impairment underwent a BOLD-CVR examination. Similarly, neurologically more affected patients, restless or anxious patients were rather not invited for BOLD fMRI study, which means the sample is probably different from the population with respect to its demographics and health outcomes. With BOLD-CVR examination a volunteer bias was introduced too, since patients had to agree with study participation and sign an informed consent. Contrary, examination of collateral status with TCD was performed in clinical setting and therefore done by several board-certified stroke neurologists, introducing additional subjectivity to already operator-dependent examination. Moreover, no re-TCDs were performed to control the findings. TCD has methodological limitations to account for leptomeningeal collateral flow – notably TCD cannot assess ACA to MCA as major contributories for total leptomeningeal collateral flow. The aim, however, was to assess TCD-derived leptomeningeal collateral activation which can be assessed from the posterior circulation.^4,6,9,21^ Moreover, using TCD we cannot assess all external to internal carotid artery connections through facial, maxillary, middle meningeal, and occipital arteries. Nonetheless from a clinical point of view, TCD is considered a useful tool to screen for collateral activation, based on four main collateral pathways (2 primary, and 2 secondary) that can be reliably assessed with TCD.^4,6,9,21^

## Conclusions

In our study, TCD-derived ipsilateral leptomeningeal collateral activation is the sole collateral pathway associated with impaired BOLD-CVR in patients with symptomatic unilateral ICA occlusion.

## Supplemental Material

sj-pdf-1-jcb-10.1177_0271678X211024373 - Supplemental material for Leptomeningeal collateral activation indicates severely impaired cerebrovascular reserve capacity in patients with symptomatic unilateral carotid artery occlusion Click here for additional data file. Supplemental material, sj-pdf-1-jcb-10.1177_0271678X211024373 for Leptomeningeal collateral activation indicates severely impaired cerebrovascular reserve capacity in patients with symptomatic unilateral carotid artery occlusion by Martina Sebök, Christiaan Hendrik Bas van Niftrik, Niklas Lohaus, Giuseppe Esposito, Mohamad El Amki, Sebastian Winklhofer, Susanne Wegener, Luca Regli and Jorn Fierstra in Journal of Cerebral Blood Flow & Metabolism

## References

[1] Flaherty ML Flemming KD McClelland R , et al. Population-based study of symptomatic internal carotid artery occlusion: incidence and long-term follow-up. Stroke 2004; 35: e349-52. 10.1161/01.STR.0000135024.54608.3f 15232124

[2] Reinhard M Schwarzer G Briel M , et al. Cerebrovascular reactivity predicts stroke in high-grade carotid artery disease. Neurology 2014; 83: 1424–1431. 25217057 10.1212/WNL.0000000000000888 PMC4206163

[3] Papassin J Heck O Condamine E , et al. Impaired cerebrovascular reactivity is associated with recurrent stroke in patients with severe intracranial arterial stenosis: a C02 BOLD fMRI study. J Neuroradiol 2020; S0150-9861(20)30165-6. 10.1016/j.neurad.2020.04.005 32466863

[4] Müller M, andSchimrigk K. Vasomotor reactivity and pattern of collateral blood flow in severe occlusive carotid artery disease. Stroke 1996; 27: 296–299. 8571426 10.1161/01.str.27.2.296

[5] Schomer DF Marks MP Steinberg GK , et al. The anatomy of the posterior communicating artery as a risk factor for ischemic cerebral infarction. N Engl J Med 1994; 330: 1565–1570. 8177246 10.1056/NEJM199406023302204

[6] Schneider J Sick B Luft AR , et al. Ultrasound and clinical predictors of recurrent ischemia in symptomatic internal carotid artery occlusion. Stroke 2015; 46: 3274–3276. 26382172 10.1161/STROKEAHA.115.011269

[7] Grubb RL Jr Derdeyn CP Fritsch SM , et al. Importance of hemodynamic factors in the prognosis of symptomatic carotid occlusion. Jama 1998; 280: 1055–1060. 9757852 10.1001/jama.280.12.1055

[8] Bonow RH Young CC Bass DI , et al. Transcranial doppler ultrasonography in neurological surgery and neurocritical care. Neurosurg Focus 2019; 47: E2. 10.3171/2019.9.FOCUS19611 31786564

[9] Liebeskind DS. Collateral circulation. Stroke 2003; 34: 2279–2284. 12881609 10.1161/01.STR.0000086465.41263.06

[10] Liebeskind DS. Collaterals in acute stroke: beyond the clot. Neuroimaging Clin N Am 2005; 15: 553–573. 16360589 10.1016/j.nic.2005.08.012

[11] Hofmeijer J Klijn CJ Kappelle LJ , et al. Collateral circulation via the ophthalmic artery or leptomeningeal vessels is associated with impaired cerebral vasoreactivity in patients with symptomatic carotid artery occlusion. Cerebrovasc Dis 2002; 14: 22–26. 12097847 10.1159/000063719

[12] van Niftrik CHB Piccirelli M Bozinov O , et al. Iterative analysis of cerebrovascular reactivity dynamic response by temporal decomposition. Brain Behav 2017; 7: e00705. 28948064 10.1002/brb3.705 PMC5607533

[13] Sebok M van Niftrik CHB Piccirelli M , et al. BOLD cerebrovascular reactivity as a novel marker for crossed cerebellar diaschisis. Neurology 2018; 91: e1328–e1337. 30185447 10.1212/WNL.0000000000006287

[14] von Bieberstein L van Niftrik CHB Sebök M , et al. Crossed cerebellar diaschisis indicates hemodynamic compromise in ischemic stroke patients. Transl Stroke Res 2021; 12: 39–48. 32506367 10.1007/s12975-020-00821-0 PMC7803723

[15] Barnett HJM Taylor DW Haynes RB , et al. Beneficial effect of carotid endarterectomy in symptomatic patients with high-grade carotid stenosis. N Engl J Med 1991; 325: 445–453. 1852179 10.1056/NEJM199108153250701

[16] Slessarev M Han J Mardimae A , et al. Prospective targeting and control of end-tidal CO2 and O2 concentrations. J Physiol 2007; 581: 1207–1219. 17446225 10.1113/jphysiol.2007.129395 PMC2170842

[17] van Niftrik CHB Piccirelli M Bozinov O , et al. Impact of baseline CO2 on blood-oxygenation-level-dependent MRI measurements of cerebrovascular reactivity and task-evoked signal activation. Magn Reson Imaging 2018; 49: 123–130. 29447850 10.1016/j.mri.2018.02.002

[18] von Reutern GM Goertler MW Bornstein NM , et al. Grading carotid stenosis using ultrasonic methods. Stroke 2012; 43: 916–921. 22343647 10.1161/STROKEAHA.111.636084

[19] Baumgartner RW. Intracranial stenoses and occlusions, and circle of willis collaterals. Front Neurol Neurosci 2006; 21: 117–126. 17290131 10.1159/000092394

[20] Baumgartner RW Baumgartner I Mattle HP , et al. Transcranial color-coded duplex sonography in unilateral flow-restrictive extracranial carotid artery disease. AJNR 1996; 17: 777–783. 8730200 PMC8337261

[21] Reinhard M Müller T Guschlbauer B , et al. Dynamic cerebral autoregulation and collateral flow patterns in patients with severe carotid stenosis or occlusion. Ultrasound Med Biol 2003; 29: 1105–1113. 12946513 10.1016/s0301-5629(03)00954-2

[22] Anzola GP Gasparotti R Magoni M , et al. Transcranial doppler sonography and magnetic resonance angiography in the assessment of collateral hemispheric flow in patients with carotid artery disease. Stroke 1995; 26: 214–217. 7831690 10.1161/01.str.26.2.214

[23] Core Team R. R: a language and environment for statistical computing. Austria: R Foundation for Statistical Computing V, 2020.

[24] Armstrong RA. When to use the Bonferroni correction. Ophthalmic Physiol Opt 2014; 34: 502–508. 24697967 10.1111/opo.12131

[25] Klijn CJ Kappelle LJ van Huffelen AC , et al. Recurrent ischemia in symptomatic carotid occlusion: prognostic value of hemodynamic factors. Neurology 2000; 55: 1806–1812. 11134377 10.1212/wnl.55.12.1806

[26] Persoon S Luitse MJ de Borst GJ , et al. Symptomatic internal carotid artery occlusion: a long-term follow-up study. J Neurol Neurosurg Psychiatry 2011; 82: 521–526. 20884678 10.1136/jnnp.2010.208330

[27] Barnett HJ Taylor DW Eliasziw M , et al. Benefit of carotid endarterectomy in patients with symptomatic moderate or severe stenosis. North American symptomatic carotid endarterectomy trial collaborators. N Engl J Med 1998; 339: 1415–1425. 9811916 10.1056/NEJM199811123392002

[28] Klijn CJ Kappelle LJ Algra A , et al. Outcome in patients with symptomatic occlusion of the internal carotid artery or intracranial arterial lesions: a meta-analysis of the role of baseline characteristics and type of antithrombotic treatment. Cerebrovasc Dis 2001; 12: 228–234. 11641588 10.1159/000047708

[29] Goode SD Altaf N Munshi S , et al. Impaired cerebrovascular reactivity predicts recurrent symptoms in patients with carotid artery occlusion: a hypercapnia BOLD fMRI study. AJNR 2016; 37: 904–909. 27012300 10.3174/ajnr.A4739 PMC7960300

[30] Ogasawara K Ogawa A Yoshimoto T. Cerebrovascular reactivity to acetazolamide and outcome in patients with symptomatic internal carotid or Middle cerebral artery occlusion: a xenon-133 single-photon emission computed tomography study. Stroke 2002; 33: 1857–1862. 12105366 10.1161/01.str.0000019511.81583.a8

[31] Failure of extracranial-intracranial arterial bypass to reduce the risk of ischemic stroke Results of an international randomized trial. N Engl J Med 1985; 313: 1191–1200. 2865674 10.1056/NEJM198511073131904

[32] Garrett MC Komotar RJ Merkow MB , et al. The extracranial-intracranial bypass trial: implications for future investigations. Foc 2008; 24: E4. 10.3171/FOC/2008/24/2/E4 18275299

[33] Hartkamp NS Petersen ET Chappell MA , et al. Relationship between haemodynamic impairment and collateral blood flow in carotid artery disease. J Cereb Blood Flow Metab 2018; 38: 2021–2032. 28776469 10.1177/0271678X17724027 PMC6238174

[34] Liu S Luo Y Wang C , et al. Combination of plaque characteristics, pial collaterals, and hypertension contributes to misery perfusion in patients with symptomatic middle cerebral artery stenosis. J Magn Reson Imaging 2020; 51: 195–204. 31069889 10.1002/jmri.26778

[35] Kim SJ Son JP Ryoo S , et al. A novel magnetic resonance imaging approach to collateral flow imaging in ischemic stroke. Ann Neurol 2014; 76: 356–369. 24985162 10.1002/ana.24211

[36] Azizyan A Sanossian N Mogensen MA , et al. Fluid-attenuated inversion recovery vascular hyperintensities: an important imaging marker for cerebrovascular disease. AJNR 2011; 32: 1771–1775. 21051516 10.3174/ajnr.A2265 PMC7966020

[37] Shuaib A Butcher K Mohammad AA , et al. Collateral blood vessels in acute ischaemic stroke: a potential therapeutic target. Lancet Neurol 2011; 10: 909–921. 21939900 10.1016/S1474-4422(11)70195-8

[38] Vernieri F Pasqualetti P Matteis M Passarelli F , et al. Effect of collateral blood flow and cerebral vasomotor reactivity on the outcome of carotid artery occlusion. Stroke 2001; 32: 1552–1558. 11441200 10.1161/01.str.32.7.1552

[39] Zhao H Wang B Xu G , et al. Collateral grade of the willis' circle predicts outcomes of acute intracranial internal carotid artery occlusion before thrombectomy. Brain Behav 2019; 9: e01452. 31696661 10.1002/brb3.1452 PMC6908856

[40] van Everdinge KJ Visser GH Klijn CJM , et al. Role of collateral flow on cerebral hemodynamics in patients with unilateral internal carotid artery occlusion. Ann Neurol 1998; 44: 167–176. 9708538 10.1002/ana.410440206

[41] Connolly F Röhl JE Lopez-Prieto J , et al. Pattern of activated pathways and quality of collateral status in patients with symptomatic internal carotid artery occlusion. Cerebrovasc Dis 2019; 48: 244–250. 31846978 10.1159/000504663

[42] Smith HA Thompson-Dobkin J Yonas H , et al. Correlation of xenon-enhanced computed tomography-defined cerebral blood flow reactivity and collateral flow patterns. Stroke 1994; 25: 1784–1787. 8073458 10.1161/01.str.25.9.1784

[43] Seiler A Brandhofe A Gracien R-M , et al. DSC perfusion-based collateral imaging and quantitative T2 mapping to assess regional recruitment of leptomeningeal collaterals and microstructural cortical tissue damage in unilateral steno-occlusive vasculopathy. J Cereb Blood Flow Metab 2021; 41: 67–81. 31987009 10.1177/0271678X19898148 PMC7747159

[44] Strother MK Anderson MD Singer RJ , et al. Cerebrovascular collaterals correlate with disease severity in adult North American patients with moyamoya disease. AJNR 2014; 35: 1318–1324. 24651814 10.3174/ajnr.A3883 PMC4367488

[45] Sam K Small E Poublanc J , et al. Reduced contralateral cerebrovascular reserve in patients with unilateral steno-occlusive disease. Cerebrovasc Dis 2014; 38: 94–100. 25277683 10.1159/000362084

[46] Viticchi G Falsetti L Buratti L , et al. Carotid occlusion: impact of cerebral hemodynamic impairment on cognitive performances. Int J Geriatr Psychiatry 2021; 36: 197–206. 32857468 10.1002/gps.5414

[47] Pavol MA Sundheim K Lazar RM , et al. Cognition and quality of life in symptomatic carotid occlusion. J Stroke Cerebrovasc Dis 2019; 28: 2250–2254. 31171458 10.1016/j.jstrokecerebrovasdis.2019.05.007 PMC6679762

