# Supplementary material for: Leptomeningeal collateral activation indicates severely impaired cerebrovascular reserve capacity in patients with symptomatic unilateral carotid artery occlusion
Source: J Cereb Blood Flow Metab. 2021 Jun 10;41(11):3039–51. doi: 10.1177/0271678X211024373 (PMC8545056; doi:10.1177/0271678X211024373)
Supplement: sj-pdf-1-jcb-10.1177_0271678X211024373 - Supplemental material for Leptomeningeal collateral activation indicates severely impaired cerebrovascular reserve capacity in patients with symptomatic unilateral carotid artery occlusion [file sj-pdf-1-jcb-10.1177_0271678X211024373.pdf]

Supplemental files

Supplementary Table

Supplementary Table 1. Baseline TCD values

Duplex parameter (in cm/s) (median (interquartile range))	Symptomatic unilateral ICA occlusion cohort
**PSV OA ipsilateral**	32 (19)
**EDV OA ipsilateral**	10 (13)
**PSV OA contralateral**	33 (17)
**EDV OA contralateral**	7 (4)
**PSV ACA ipsilateral**	66 (52)
**EDV ACA ipsilateral**	27 (22)
**PSV ACA contralateral**	101 (65)
**EDV ACA contralateral**	45 (30)
**PSV M1 ipsilateral**	76 (45)
**EDV M1 ipsilateral**	34 (20)
**PSV M1 contralateral**	114 (43)
**EDV M1 contralateral**	44 (20)
**PSV P1 ipsilateral**	84 (46)
**EDV P1 ipsilateral**	33 (22)
**PSV P1 contralateral**	70 (29)
**EDV P1 contralateral**	26 (13)
**PSV P2 ipsilateral**	67 (43)
**EDV P2 ipsilateral**	27 (16)
**PSV P2 contralateral**	55 (31)
**EDV P2 contralateral**	23 (12)

ACA=anterior cerebral artery, EDV=end diastolic velocity, ICA=internal carotid artery, M1=first segment of middle cerebral artery, OA=ophthalmic artery, PSV=peak systolic velocity, P1=first segment of posterior cerebral artery, P2=second segment of posterior cerebral artery
*ipsilateral = on the side of occluded ICA, **contralateral = opposite side of occluded ICA
